# Effect of dietary heated hemp seed cake and phytase as soybean meal substitution on broiler chicken performance, carcass yield, visceral organ weight, intestinal health, and serum biochemical parameters

**DOI:** 10.1007/s11250-025-04416-5

**Published:** 2025-04-08

**Authors:** Arif Darmawan, Ergin Ozturk

**Affiliations:** 1 Department of Animal Nutrition and Feed Technology, Faculty of Animal Science, IPB University, Bogor, Indonesia; 2 Department of Animal Science, Faculty of Agriculture, Ondokuz Mayis University, Samsun, Türkiye

**Keywords:** Anti-nutrients, Body weight, Enzyme, Gut microbiota, Maillard

## Abstract

Hemp seed is a potential candidate to replace soybean meal dependency due to its rich protein and balanced amino acid profile content despite containing anti-nutrients. This study purposed to evaluate dietary heated hemp seed cake (HSC) and the addition of phytase as a substitution for soybean meal on broiler productive performances, carcass characteristics, visceral organ weight, serum biochemical and intestinal health of broiler chickens. A total of 210 unsexed Ross broiler chicks were randomly divided into 7 treatments and 6 replicates using a completely randomized design and reared for 42 days. The treatments were T1: Corn-soybean-based ration; T2: Ration containing 15% unheated HSC; T3:T2 + 1000 FTU of phytase; T4: Ration containing 15% autoclaved HSC (120 °C,10 min); T5: T4 + 1000 FTU of phytase; T6: Ration containing 15% oven-heated HSC (120 °C,20 min); T7: T6 + 1000 FTU of phytase. On day 42, the body weight and body weight gain of the T4 group were higher (*P* < 0.01) compared to the T2, T3, T6, and T7 groups. The relative length of the jejunum, ileum, and cecum in all HSC diets was higher than that of the T1 group (*P* < 0.01). Dietary T4 lowered total bacteria and *E. coli* and enhanced *L. acidophilus* population in the cecum (*P* < 0.01). The villus height and surface area in all HSC diets were lower than those in the T1 group (*P* < 0.01). Glucose and calcium levels in the T4 and T5 groups were higher than those in the T1, T2, and T3 groups (*P* < 0.01). It concluded that dietary autoclaved HSC has positive effects on the gut health and growth of broiler chickens compared to dietary oven-heated HSC and unheated HSC. However, the positive effects cannot match those of soybean meal and corn-based diets.

## Introduction

Soybean meal is an important poultry feed ingredient due to its high protein content and balanced amino acids, but price fluctuations pose challenges to its use. Hemp seed cake (HSC) has emerged as a potential alternative with high protein, polyunsaturated fatty acids, and minerals, although limited by anti-nutritional factors (Öztürk et al. 2024). The dominant anti-nutrients in hemp seeds are phytic acid (22.5 mg/g), cyanogenic glycosides (3.8 µmol/g), trypsin inhibitors (2.88 TIU/mg), and tannins (0.23 mg/g) (Russo and Reggiani 2015). Hemp seed contains 35% oil, 25% crude protein, 30% carbohydrates, 29%−37% crude fiber, tocopherols, and flavonols and is rich in minerals such as magnesium, phosphorus, iron, and calcium. In addition, the fatty acids in hemp seed reach 65–80% polyunsaturated fatty acid, with the main proportion of linolenic acid and linoleic acid. It is even a great source of unsaturated fatty acids compared to soybean oil, with an omega-3 to omega-6 ratio of 3.3 (Wang and Xiong 2019; Semwogerere et al. 2020; Feng et al. 2022).

Previous studies showed that HSC could be utilized in broiler diets with varying results depending on the level of use due to its anti-nutrients and crude fiber content. The inclusion of 10% HSC (Tufarelli et al. 2023) and 7.5% hemp seed containing 0.1% dextran oligosaccharide (Khan et al. 2010) did not affect live weight and feed efficiency. However, 5% HSC could be used in broiler diets as a substitute for soybean meal, while 15% HSC reduced the live weight of broilers (Ondrej et al. 2015; Šťastník et al. 2019). Therefore, pretreatment in the use of high-level HSC is needed to reduce the levels of antinutrients, such as the utilization of exogenous enzymes and the heating process. The positive effect of utilizing phytase enzyme was reported by Ptak et al. (2015) that Ca and P released through phytate hydrolysis improved the proliferation of lactic acid bacteria and broiler performance. Furthermore, hydrolyzing phytate and minerals can improve bone and gut morphology health (Krieg et al. 2021). Regarding the effect of heating treatments, it was reported that oven-heated tiger nuts at 120 °C for 30 min decreased the content of carbohydrates, tannin, phytate, alkaloid, and oxalate (Adekanmi et al. 2009). Heated HSC at 120 °C for 60 min resulted in better egg quality and laying hen productive performance compared to raw HSC (Konca et al. 2019). However, increasing the temperature and heating time (128 °C for 20 min) also decreased the protein and starch content (Rehman and Shah 2005).

Despite the previously discussed benefits of heat treatment and phytase enzyme on broiler performance, it is imperative to further assess carcass quality, serum parameters, visceral organs, and intestinal morphology with microflora to determine production yield, chicken health, and gastrointestinal tract health. Notably, evaluation of the combination effect of high-level HSC (15%) with phytase has not been reported previously and is essential to optimize its utilization and poultry performance. This study hypothesized that heating treatment could reduce the anti-nutrients of HSC, and its use with phytase in broiler diets could improve gut and chicken health, balance gut microbiota, and ultimately improve broiler performance. Thus, our study purposed to evaluate dietary heated HSC and the addition of phytase enzyme as a substitution for soybean meal on broiler productive performances, carcass, visceral organs weight, serum biochemical, and intestinal health of broiler chickens.

## Materials and methods

### Animals and rearing management

All procedures in the current study were performed according to Ondokuz Mayis University Ethics Committee guidelines (No: 57/2022). A total of 210 unsexed Ross broiler chicks were obtained from a commercial hatchery (Ross Breeders Anadolu, Turkiye). The study employed a completely randomized design with seven treatments and six replicates. The chickens were randomly divided into 42 partitioned cages (1 m × 1.15 m × 0.7 m) and were reared for 42 days. The ambient temperature was set at 33^0^ C for 2 days of rearing and reduced by 1 °C gradually until it reached 20 °C in the finisher phase. Lighting was 24 h from the arrival of the chicks until 3 days, then changed to 23 h of light and 1 h of dark. Gumboro vaccine was conducted on day 21, and Newcastle Disease on day 28.

### Diets and treatments

Diets were prepared based on the starter (1–10 days), grower (11–21 days), and finisher (22–42 days) periods (Table 2). The diet was formulated according to the nutritional requirements of Ross strain broilers. The metabolizable energy, crude protein, crude fat, amino acid, and mineral contents of the diet were determined by calculation, while the crude fiber was analyzed using method 973.18 (AOAC 1990). Mash diet and drinking water were provided ad libitum. The treatments were:

- T1: Corn-soybean-based basal ration.
- T2: Ration containing 15% unheated HSC.
- T3: Ration containing 15% unheated HSC and 1000 FTU of phytase.
- T4: Ration containing 15% autoclaved HSC (120 °C,10 min).
- T5: Ration containing 15% autoclaved HSC (120 °C,10 min) and 1000 FTU of phytase.
- T6: Ration containing 15% oven-heated HSC (120 °C,20 min).
- T7: Ration containing 15% oven-heated HSC (120 °C,20 min) and 1000 FTU of phytase.

### Preparation of HSC

Vezir local hemp seeds from the Cannabis Research Center, Ondokuz Mayis University, were cold-pressed to obtain HSC. The pelletized HSCs were ground into a mash before being heated using an autoclave and oven. Then, HSC was heated in the oven at 120 °C for 20 min and autoclaved at 120 °C for 10 min. The heated and unheated HSC were analyzed for moisture content and proximate using the AOAC (1990) method, as well as anti-nutrients (phytic acid, tannin, HCN, saponin) using the method described by Russo and Reggiani (2015). The nutrient composition and anti-nutrients of HSC are provided in Table 1 and Fig. 2.

**Table 1:** Nutrient composition of HSCs used in the study

Treatments	Dry Matter (%)	Ash (%)	Crude protein (%)	Extract ether (%)
Unheated HSC	91.49	6.44	36.87	16.60
Outoclved HSC	91.25	6.35	36.87	16.00
Oven-heated HSC	94.03	6.67	37.28	16.78

### Broiler chicken performances

The performance of broiler chickens was evaluated up to 42 days of age. The initial DOC weight was recorded upon arrival. Body weight and remaining feed were weighed weekly. Feed intake was calculated by subtracting the remaining feed from the amount of feed provided. Mortality was recorded daily. The feed conversion ratio was determined by dividing the feed intake by the body weight gain.

### Carcass and visceral organ

At the age of 42 days, the chickens were fasted overnight and provided with access to drinking water. Subsequently, all chickens within each replicate were individually weighed, and one chicken was selected for slaughter with a weight close to the average weight of chickens per replicate. The chicken was immediately de-feathered, and the visceral organs were excluded. The carcass, gizzard, heart, liver, spleen, abdominal fat, and intestine were weighed with an accuracy of 0.1 g using a digital scale. The visceral organ weight percentage was determined by dividing the organ weight by the live weight and then multiplying it by 100. Additionally, the intestinal length was measured with a measuring tape and expressed as cm/kg live weight.

### Cecum microflora population

At the age of 42 days, cecum samples were collected during dissection from 1 chicken and stored at −21 °C until the bacterial population was counted. Analysis was performed as described according to Erener et al. (2020). A total of 1 g sample was homogenized with 0.9% normal saline. Tenfold serial dilutions of each sample were prepared in sterile Ringer's solution. 100 µL of each dilution was spread on specific media such as plate count agar for the total bacterial count after incubation at 30 °C for 48 h, coliform agar for coliform bacteria after incubation at 37 °C for 48 h, de Man Rogosa agar, and Sharpe for *Lactobacillus* spp. after incubation at 30 °C for 72 h, Eosin Methylene Blue agar for *E. coli* at 35 °C for 24 h. Microbial counts were converted to log10 CFU/g.

### Jejunum morphology

At the age of 42 days, one broiler chicken from each replication was selected based on the weight being the same or close to the average broiler weight. The selected chickens were then slaughtered to measure intestinal morphology. Jejunum samples were taken immediately after slaughter and immersed in 10% neutral buffered formalin. Two-centimeter jejunum samples were dissected and dehydrated by adding ethyl alcohol at concentrations of 70%, 90%, 96%, and 100%. Then, it was washed in xylene and immersed in paraffin. Tissues were cut into 6 µm thick sections using a microtome and were attached to a glass slide and colored with hematoxylin–eosin. The height, basal width, and apical width of the villus were calculated using an Olympus microscope at 4 times magnification and were captured by a video microscope (video gauge IV-560). The villus area was calculated from the villus height, apical width, and basal width. The depth of the crypt was measured as the distance from the base of the villus to the muscle layer. The villus surface area was calculated using the following formula (Iji et al. 2001).

$$\mathrm{Villi}\;\mathrm{Surface}\;\mathrm{area}\;\left(\mathrm{\mu m}^2\right):\;(\mathrm B+\mathrm C)/\mathrm C\;\times\;\mathrm A$$

A: villus height
B: basal width
C: apical width

### Serum biochemical parameters

At 42 days of age, 2 ml blood samples were collected from the wing vein of the bird using a disposable syringe from 1 broiler per replicate and transferred into a serum separator tube containing gel. The blood sample was centrifuged (3500 g,10 min,4 °C) to obtain serum (Kumar et al. 2020). The concentrations of triglyceride, cholesterol, total protein, albumin, creatinine, glucose, bilirubin, phosphorus, calcium, gamma-glutamyl transferase, alkaline phosphatase, aspartate aminotransferase were determined using commercial kits in a quality-certified laboratory.

### Statistical analysis

The evaluation of data normality was tested with the Shapiro–Wilk test. Data analysis was performed in a completely randomized design with one-way Analysis of Variance (ANOVA) using the IBM SPSS Statistics 22 program (Chicago, IL, USA). Subsequently, the significance values at *P* < 0.05 were subjected to further testing using the Duncan Multiple Range Test. Bacterial counts were log10-transformed before statistical analysis. All data were presented as mean with a standard error of the mean (SEM).

## Results

### Broiler chicken performance

The productive performances of broiler chickens are presented in Table 2. On day 21, the body weight of the T4 group was higher compared to the T2, T5, T6, and T7 groups (*P* < 0.01). On day 42, the body weight of the T4 group was higher compared to the T2, T3, T6, and T7 groups but lower than that of the T1 group (*P* < 0.01). The T2 group produced the lowest body weight.

**Table 2:** The composition and nutrient content of broiler chicken diets (*as fed*)

Ingredients (%,)	Starter (1–10 days)		Grower (11–21 days)		Finisher (22–42 days)	
	Control	HSC	Control	HSC	Control	HSC
Maize	53.20	48.31	59.00	54.00	65.18	60.20
HSC (37%)	0	15.00	0	15.00	0	15.00
Soybean meal (44%)	39.50	29.00	34.50	24.00	29.00	18.50
Vegetable oil	3.15	4.00	3.20	4.00	2.80	3.60
Dicalcium phosphate	2.17	2.12	1.76	1.68	1.42	1.39
Limestone	0.80	0.80	0.58	0.60	0.56	0.56
Dl-Methionine	0.31	0.27	0.27	0.23	0.26	0.22
Nacl	0.37	0.37	0.37	0.37	0.37	0.37
Premix*	0.10	0.10	0.10	0.10	0.10	0.10
L-Lysine sulfate	0.22	0.02	0.18	0.01	0.27	0.05
L-Threonine	0.18	0.01	0.04	0.01	0.04	0.01
Total	100.00	100.00	100.00	100.00	100.00	100.00
Nutrient content (based on calculation)						
Metabolizable energy (MJ/kg)	12.48	12.47	12.81	12.79	13.02	12.99
Crude protein (%)	23.50	23.50	21.50	21.60	19.50	19.50
Crude fat (%)	3.60	4.01	3.80	4.30	3.06	4.06
Lysine (%)	1.32	1.32	1.18	1.19	1.10	1.08
Methionine + cystine (%)	1.00	1.00	0.92	0.92	0.86	0.86
Available phosphorus (%)	0.50	0.5	0.43	0.42	0.36	0.36
Calcium (%)	0.95	0.95	0.78	0.76	0.65	0.66

^*^Premix composition per kg of feed: 3 000 UI cholecalciferol, 12 000 IU retinol, 60 mg α-tocopherol, 4 mg menadione, 10 mg riboflavin, 3 mg thiamine, 50 mg niacin, 4 mg pyridoxine,14 mg Ca-D-pantothenate, 0. 030 mg cyanocobalamin, 0.25 mg biotin, 250 mg choline chloride, 2 mg folic acid, 15 mg Cu, 120 mg Mn, 50 mg Fe, 1.5 mg I, 100 mg Zn, 0.3 mg Se, 0.1 mg Co

On day 21, groups T3 and T4 yielded higher body weight gain compared to T2, T5, T6, and T7 groups (*P* < 0.01). On days 22–42, the T4 group had higher body weight gain compared to the T2, T3, and T6 groups (*P* < 0.01). In the overall 42-day period, the T4 group yielded higher body weight gain than the T2, T3, T6, and T7 groups but lower than the T1 group (*P* < 0.01). The T2 group produced the lowest body weight gain.

On day 42, the highest feed intake was produced by a group of T2. The inclusion of HSC and phytase (T2-T7) had a higher FCR compared to the T1 group (*P* < 0.01).

#### Carcass and visceral organ weight

The treatments had no significant effect on the weight of the carcass, liver, abdominal fat, duodenum, cecum, and duodenum length (Table 3). Dietary in all HSC diets produced a higher percentage of heart weight compared to dietary T1 (*P* = 0.02), and T2 and T3 groups had the highest percentage of spleen weight (*P* = 0.04) and had a higher percentage of gizzard weight compared to T1 and T5 groups (*P* = 0.01). Dietary T2, T4, T5, T6, and T7 produced a higher percentage of jejunum weight compared to dietary T1 (*P* < 0.01). Dietary T2, T3, T6, T6, and T7 produced a higher percentage of ileum weight compared to dietary T1 (*P* < 0.01). The relative length of the jejunum, ileum, and cecum in all HSC diets was higher than that of the T1 group (*P* < 0.01).

**Table 3:** Effect of dietary HSC and phytase on the productive performance of broiler chicken

Parameter	Day	T1	T2	T3	T4	T5	T6	T7	SEM	*P*.value
Body weight (g/bird)	1	42.27	43.73	43.79	43.58	43.06	42.78	42.88	0.17	0.19
	21	945^**d**^	805^**a**^	830^**bc**^	840^**c**^	806^**a**^	814^**ab**^	810^**a**^	7.38	< 0.01
	42	2935^**d**^	2355^**a**^	2466^**b**^	2614^**c**^	2520^**bc**^	2438^**b**^	2490^**b**^	29.6	< 0.01
Body weight gain (g/bird)	1–21	902^**a**^	761^**c**^	786^**b**^	796^**b**^	763^**c**^	771^**c**^	767^**c**^	7.43	< 0.01
	22–42	19901^**a**^	1550^**d**^	1636^**cd**^	1774^**b**^	1714^**bc**^	1624^**cd**^	1680^**bc**^	23.75	< 0.01
	1–42	2893^**a**^	2311^**d**^	2421^**c**^	2571^**b**^	2477^**bc**^	2395^**cd**^	2447^**c**^	29.8	< 0.01
Feed intake (g/bird)	1–21	1250	1289	1238	1286	1261	1206	1217	9.36	0.12
	22–42	3502^c^	3052^a^	3080^ab^	3244^b^	3287^b^	3259^b^	3187^b^	27.93	< 0.01
	1–42	4752^c^	4317^a^	4341a^b^	4531^b^	4549^b^	4466^b^	4405^b^	26.7	< 0.01
Feed conversion ratio	1–21	1.39^a^	1.69^**b**^	1.58^**b**^	1.62^**b**^	1.65^**b**^	1.57b	1.59^**b**^	0.017	< 0.01
	22–42	1.76^a^	1.97^b^	1.88^**ab**^	1.83^ab^	1.92^ab^	2.01^**b**^	1.90^**ab**^	0.021	0.02
	1–42	1.64^a^	1.88^b^	1.78^b^	1.76^b^	1.84^b^	1.86^b^	1.80^**b**^	0.016	< 0.01
Mortality	1–42	0	0	0	0	0	0	0		

*SEM* Standard error of the mean, *T1* Corn-soybean-based basal ration, *T2* Ration containing 15% unheated HSC, *T3* Ration containing 15% unheated HSC and 1000 FTU of phytase, *T4* Ration containing 15% autoclaved HSC (120°C, 10 min), *T5* Ration containing 15% autoclaved HSC (120°C, 10 min) and 1000 FTU of phytase, *T6* Ration containing 15% oven-heated HSC (120°C, 20 min), *T7* Ration containing 15% oven-heated HSC (120°C, 20 min) and 1000 FTU of phytase. Different superscripts in the same row are significantly different at *P* < 0.05 and very significantly different at *P* < 0.01. Each value is the mean of 6 replicates of each treatment (5 birds per replicate)

### Cecum microflora and jejunum morphology

Total coliform did not change with dietary treatments (Table 5). Dietary T4 resulted in a significant decrease in total bacteria and *E. coli* and an increase in the *L. acidophilus* population in the cecum (*P* < 0.01). In addition, the addition of phytase (T3, T5, T7) had lower total bacteria and *E. coli* populations compared to dietary HSC without phytase (T2, T4, T6) (*P* < 0.01).

Crypt depth and villus width were not affected by dietary treatments (Table 6). The villus height and surface area in all HSC diets were lower than those in the T1 group (*P* < 0.01). However, the villus height in the T4 group was significantly higher compared with the T2 and T3 groups (*P* < 0.01).

### Serum biochemical parameters

The treatment did not affect cholesterol, total bilirubin, creatinine, phosphorus, and alkaline phosphatase levels (Table 7). However, the T6 and T7 diets significantly increased (*P* < 0.01) serum triglycerides and gamma-glutamyl transferase, and the T7 group had higher aspartate aminotransferase levels compared to the T1, T4, and T5 groups. Glucose and calcium levels in groups T4 and T5 were higher than those in the T1, T2, and T3 groups (*P* < 0.01). However, dietary T2 significantly increased total protein compared to T1, T5, T6 and T7 groups (*P* < 0.01).

## Discussion

### Broiler chicken performance

**Fig. 1:**
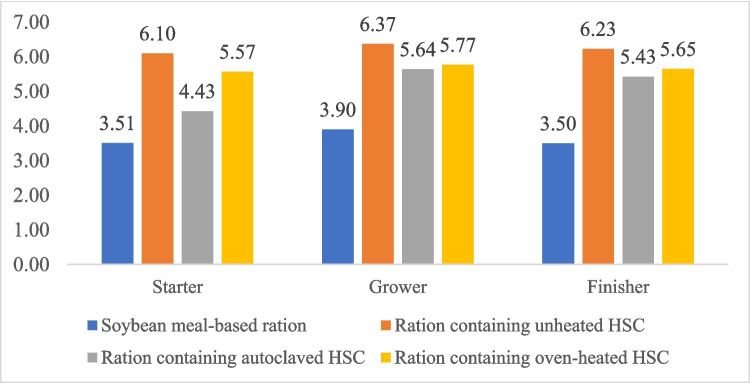
Percentage of crude fiber (%) of starter phase, growth phase, and finisher phase diets containing soybean meal, unheated HSC, and heated HSC

In our study, the weight of broiler chickens decreased when high levels of HSC (15%) were used in the diet. Furthermore, the heating of HSC and adding the phytase enzyme to the diet were also unable to match the broiler chickens' performances fed a corn-soya bean-based diet. The decrease in body weight and body weight gain in our study was caused by the low consumption of broiler chickens in all HSC groups, which also had an impact on increasing FCR. In addition, the adverse effect on body weight with HSC treatment may be due to the high fiber content in the feed. The crude fiber of starter, grower, and finisher rations containing 15% HSC reached 6.10%, 6.37%, and 6.23%, respectively (Fig. 1). This content exceeds the crude fiber recommended by the National Research Council (1994) of 5%. Previously, Sittiya et al. (2020) reported that feed containing 5% crude fiber reduced the body weight of broiler chickens compared to feed with 2.5% crude fiber. The high crude fiber in the feed increases the digestive viscosity of the small intestine, further reducing feed utilization and body weight (Eizadi et al. 2015). Dietary fiber inhibits the activity of endogenous enzymes and reduces lipase and bile salt, which consequently impairs the digestion and utilization of fat (Al-Harthi et al. 2023).

**Fig. 2:**
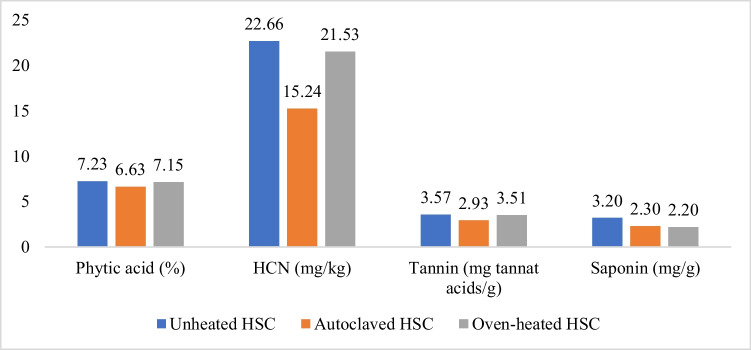
Effects of heating on antinutrient content of HSC

The results of our study indicated a positive effect of autoclaving. The autoclaved HSC treatment yielded the highest body weight among the other HSC treatments, although it did not reach the body weight of the soybean meal-based diet. The higher feed intake in the heated HSC diet group with or without phytase compared to unheated HSC was believed to contribute to the increased body weight in this study. In addition, autoclaving with humidity and pressure improved starch digestibility due to hydrolysis and gelatinization processes (Dundar and Gocmen 2013) and was demonstrated to reduce trypsin inhibitors and other anti-nutrients, enhanced nutrient digestibility, and hence improved broiler performance (Eizadi et al. 2015; Al-Harthi et al. 2023). A decrease in anti-nutrients in autoclaved HSC was also observed in this study (Fig. 2), which might have contributed to the improvement of broiler performance. Anti-nutrients can interfere with the digestion and utilization of nutrients in the digestive system by mechanisms including preventing endogenous enzyme activity through viscosity formation and decreased bile salt secretion. Anti-nutrients also bind nutrients and minerals to form a complex that cannot be absorbed by the villi. Anti-nutrients damage the structure and function of the cells of the digestive organs, causing digestive disorders and ultimately reducing nutrient absorption (Al-Harthi et al. 2023).

The addition of enzymes to the unheated HSC was able to increase performance (body weight, feed intake, body weight gain) when compared to the unheated HSC without phytase. These findings indicated that the incorporation of 1,000 IU/kg of phytase enzyme was able to mitigate the adverse effects of dietary unheated HSC. Unexpectedly, the addition of phytase to diets with heated HSCs did not help improve the performance of broiler chickens. Instead, a positive effect of phytase was noticed in the unheated HSC diet, as evidenced by higher body weights compared to the unheated HSC group without phytase. This assumption was supported by the statement of Al-Harthi et al. (2023) that there was no effect of using exogenous enzymes in autoclaved moringa seed meal due to the effectiveness of exogenous enzymes determined by the amount of antinutrient content of feed ingredients. In this study, the autoclaving effectively reduced the phytic acid of HSC (Fig. 2) Some phytic acids can be degraded by autoclaving into myoinositol phosphate and inorganic phosphate, which increases P digestibility and inhibits phytate complexes formation with other nutrients (Haetinger and Adeola 2023). Also, in this study, autoclave heating (120^0^C, 10 min) appeared more effective than oven heating (120^0^C, 20 min), as evidenced by higher body weight. This might be due to the negative effects of the long-term oven heating. According to Oliveira et al. (2019), exposure of feed ingredients to high temperatures for long durations caused a Maillard reaction that decreased amino acid digestibility and reduced the availability of vitamins and minerals.

### Carcass and visceral organ weight

In this study, the dietary HSC and phytase did not result in a significant effect on carcass weight. However, dietary HSC increased the weight of the heart, gizzard, spleen, and intestinal size, which may be due to the crude fiber composition of HSC (Table 4). According to Kasula et al. (2021), HSC is a source of crude fiber reaching 30% dry weight. In the present study, crude fiber levels of between 6.10 and 6.37% (Fig. 1) in the diet were associated with an increase in the size of several organs in broilers. The increase in gizzard size as a site for mechanical digestion appeared significant in the unheated HSC treatment. On the other hand, the heated HSC diet seemed to have a smaller gizzard size compared to the unheated HSC diet. This might be due to the reduction of crude fiber and anti-nutrients due to the heating process. Indeed, the administration of moderate amounts of insoluble fiber increased nutrient digestibility due to enhanced chyme retention in the gastrointestinal tract and the production of digestive enzymes. On the other hand, high crude fiber intake interfered with nutrient retention and reduced poultry performance since crude fiber was not able to be broken down by digestive enzymes (Zhang et al. 2023a, b).

**Table 4:** Effect of dietary HSC and phytase on the carcass and visceral organs weight of broiler chicken

Parameters	T1	T2	T3	T4	T5	T6	T7	SEM	*P*-value
	Relative weight (Percent of live weight) (%)								
Carcass	74.05	74.08	74.06	73.97	74.7	72.52	74.52	0.28	0.52
Heart	0.48^a^	0.60^b^	0.59^b^	0.58^b^	0.58^b^	0.63^b^	0.55^ab^	0.01	0.02
Liver	1.59	1.67	1.66	1.61	1.69	1.66	1.6	0.024	0.07
Gizzard	1.77^a^	2.31^b^	2.15^b^	2.08^ab^	1.75^a^	1.97^ab^	1.95^ab^	0.31	0.01
Spleen	0.09^a^	0.14^b^	0.11^b^	0.10^a^	0.09^a^	0.11^a^	0.09^a^	0.03	0.04
Abdominal fat	0.86	1.34	1.39	1.28	1.45	1.34	1.3	0.04	0.87
Duodenum	0.57	0.69	0.87	0.71	0.51	0.68	0.69	0.02	0.42
Jejunum	0.97^a^	1.59^b^	1.11^ab^	1.28^b^	1.36^b^	1.50^b^	1.32^b^	0.05	< 0.01
Ileum	0.75^a^	1.04^b^	1.23^b^	0.99^ab^	0.99^ab^	1.16^b^	1.02^b^	0.03	< 0.01
Cecum	0.49	0.49	0.59	0.47	0.54	0.5	0.44	0.02	0.21
	Relative length (cm/kg live weight)								
Duodenum	13.24	14.01	14.35	12.87	13.08	14.62	14.93	0.31	0.45
Jejunum	31.74^a^	40.58^c^	35.07^bc^	33.18^b^	35.67^bc^	37.16^bc^	35.96^b^	0.75	< 0.01
Ileum	30.66^a^	35.05^b^	34.76^b^	31.84^b^	33.86^b^	34.86^b^	35.21^b^	0.81	<0.01
Cecum	6.12^a^	8.97^b^	8.14^b^	7.61^ab^	8.33^b^	8.76^b^	8.51^b^	0.19	< 0.01

*SEM* Standard error of the mean, *T1* Corn-soybean-based basal ration, *T2* Ration containing 15% unheated HSC, *T3* Ration containing 15% unheated HSC and 1000 FTU of phytase, *T4* Ration containing 15% autoclaved HSC (120 °C, 10 min), *T5* Ration containing 15% autoclaved HSC (120 °C, 10 min) and 1000 FTU of phytase, *T6* Ration containing 15% oven-heated HSC (120 °C, 20 min), *T7* Ration containing 15% oven-heated HSC (120 °C, 20 min) and 1000 FTU of phytase. Different superscripts in the same row are significantly different at *P* < 0.05 and very significantly different at *P* < 0.01. Each value is the mean of 6 replicates of each treatment (one bird per replicate)

In this study, the small intestine (jejunum, ileum) and cecum were also enlarged by the HSC-containing diets. Naturally, crude fiber caused the digestive organs, including the small intestine, to work harder to absorb nutrients, resulting in morphological changes characterized by an enlargement of size. However, when non-starch polysaccharides could be fermented in the cecum by certain microbes, they produced short-chain fatty acids, lactic acid, CH_4_, H_2_, and CO_2_ (Jha and Mishra 2021), which might be responsible for the increase in cecum size in the present study. Meanwhile, heart size was also larger in the HSC group than in the group without HSC. According to Badaruddin et al. (2022), the presence of toxins or anti-nutrients in diets that infect the heart is a contributing factor to heart enlargement. Regarding the immune organs, enlargement of the spleen when fed unheated HSC might also be caused by the negative effect of anti-nutrients on the immune response of broiler chickens (Kuku et al. (2014).

### Cecum microflora and jejunum morphology

The combination of autoclave heating and phytase was also effective in increasing the *Lactobacillus* population compared to other treatments (Table 5). Indeed, the starch, fiber, and mineral contents of feedstuffs affect the gut microbial composition (Zhang et al. 2023a, b; Jha and Mishra 2021; Omotoso et al. 2023). Nevertheless, the specific role of autoclaving in influencing the activity and composition of bacteria in the poultry digestive tract is limited. In the present study, the increase in *Lactobacillus* bacteria in line with the decrease in the *E. coli* population is associated with the role of *Lactobacillus* bacteria in suppressing the growth of pathogenic bacteria. *Lactobacillus* bacteria are known for their ability to ferment carbohydrates, which produce lactic acid (Boroojeni et al. 2014). Another reason is related to feed hygiene due to the autoclaving process affecting the bacterial status in the digestive tract of broiler chickens (Nari et al. 2019).

**Table 5:** Effect of dietary HSC and phytase on the microbial population of the cecum (Log CFU/g) of broiler chicken

Parameter	T1	T2	T3	T4	T5	T6	T7	SEM	*P*.value
Total bacteria	6.81c	7.10e	6.92d	6.58b	6.26a	7.05e	6.78 c	0.042	< 0.01
Total coliform	4.2	4.76	4.93	4.46	4.72	4.92	4.43	0.041	0.08
*E. Coli*	4.00c	4.72f	4.62e	3.79b	3.47a	4.64ef	4.21d	0.07	< 0.01
*L.Acidophilus*	3.62a	3.98b	3.86ab	4.34d	4.16 cd	3.87ab	3.68a	0.043	< 0.01

*SEM* Standard error of the mean, *T1* Corn-soybean-based basal ration, *T2* Ration containing 15% unheated HSC, *T3* Ration containing 15% unheated HSC and 1000 FTU of phytase, *T4* Ration containing 15% autoclaved HSC (1200C, 10 min), *T5* Ration containing 15% autoclaved HSC (1200C, 10 min) and 1000 FTU of phytase, *T6* Ration containing 15% oven-heated HSC (1200C, 20 min), *T7* Ration containing 15% oven-heated HSC (1200C, 20 min) and 1000 FTU of phytase. Different superscripts in the same row are very significantly different at *P* < 0.01. Each value is the mean of 6 replicates of each treatment (one bird per replicate)

In this study, the addition of phytase increased beneficial bacteria and decreased pathogenic bacteria. Reducing undigested phytic acid in the gut has a significant impact on the gut environment. One important effect is that lowering the pH in the gut can exert a bacteriostatic effect that inhibits the proliferation of pathogenic bacteria, creates a favorable environment for beneficial bacteria growth, and can potentially increase the lactic acid bacteria population (Abun et al. 2024). Phytase enzymes are reported to positively impact gut microflora through the mechanism of inducing intestinal alkaline phosphatase activity, including dephosphorylation of bacterial lipopolysaccharides and preventing transepithelial transport of bacteria (Kriseldi et al. 2021). Previous studies reported phytase supplementation increased *Lactobacillus* sp., which was positively correlated with the production of antimicrobials, including organic acids, which were able to inhibit the colonization of pathogenic microbes (Ptak et al. 2015).

This study indicated that autoclaved HSC treatment had better villus height compared to the unheated HSC group although it still did not reach the level of the corn-soya bean-based diet (Table 6). The use of phytase has also not been able to improve intestinal morphology. This may be due to the high crude fiber content in the use of HSC that damages intestinal villi (Jha and Mishra 2021; Tajeda and Kim 2021). Ultimately, these effects impair nutrient absorption, growth performance, and overall health of broilers. On the other hand, autoclaving was proven to be able to degrade anti-nutrients and fiber (Figs. 1 and 2), thus reducing villi damage and increasing nutrient absorption as evidenced by better body weight compared to unheated HSC or oven-heated HSC.

**Table 6:** Effect of dietary HSC and phytase on the jejunum intestine morphology of broiler chickens

Parameters	T1	T2	T3	T4	T5	T6	T7	SEM	*P*.value
Crypt depth (μm)	196.29	156.06	181.04	182.21	140.5	171.76	171.14	5.053	0.06
Villus height (μm)	1170.96c	687.06a	734.94a	929.26b	790.10ab	803.74ab	772.06ab	29.54	< 0.01
Villus width (μm)	113.49	121.19	105.54	104.16	116.36	101.6	99.68	3.10	0.45
Surface area (mm^2^)	0.134a	0.084b	0.076b	0.095b	0.093b	0.082b	0.078b	0.004	< 0.01

*SEM* Standard error of the mean, *T1* Corn-soybean-based basal ration, *T2* Ration containing 15% unheated HSC, *T3* Ration containing 15% unheated HSC and 1000 FTU of phytase, *T4* Ration containing 15% autoclaved HSC (120 °C, 10 min), *T5* Ration containing 15% autoclaved HSC (120 °C, 10 min) and 1000 FTU of phytase, *T6* Ration containing 15% oven-heated HSC (120 °C, 20 min), *T7* Ration containing 15% oven-heated HSC (120 °C, 20 min) and 1000 FTU of phytase. Different superscripts in the same row are very significantly different at *P* < 0.01. Each value is the mean of 6 replicates of each treatment (one bird per replicate)

### Serum biochemical

The serum biochemical of broiler chicken fed HSC is provided in Table 7. Dietary oven-heated HSC significantly increased triglycerides and increased gamma-glutamyl transferase and aspartate aminotransferase of serum. This may be due to overheating, which leads to changes in lipid composition and structure. Indeed, HSC contains high lipids, including fatty acids and triglycerides. Lipid oxidation can occur when exposed to overheating, leading to free radicals formation and other reactive oxygen species (Geng et al. 2023). This oxidative process can result in the formation of oxidized lipids, which are known to cause lipid degradation and loss of essential fatty acids that may contribute to decreased serum triglyceride levels.

**Table 7:** Effect of dietary HSC and phytase on the serum biochemical of broiler chicken

Parameters	T1	T2	T3	T4	T5	T6	T7	SEM	*P*.value
Triglyceride	23.23a	24.81a	24.53a	24.23a	24.55a	29.99b	28.44b	0.88	< 0.01
Cholesterol	111.76	108.03	107.56	107.86	115.42	114.08	116.22	1.12	0.11
Total protein	2.93a	3.94b	3.43ab	3.41ab	2.98a	2.79a	2.76a	0.10	< 0.01
Total bilirubin	0.57	0.53	0.46	0.50	0.72	0.65	0.63	0.02	0.09
Albumin	1.23c	1.33c	1.33c	1.05ab	1.03a	1.20bc	1.03a	0.02	< 0.01
Creatinine	0.03	0.06	0.03	0.06	0.06	0.04	0.05	0.005	0.36
Glucose	182.72a	185.83a	200.66ab	235.08c	233.14c	224.65bc	197.49a	3.95	< 0.01
Phosphorus	5.76	5.92	5.35	6.13	6.08	6.11	6.27	0.01	2.11
Calcium	7.88ab	7.73ab	7.21a	8.58bc	8.93c	7.92ab	8.58bc	0.11	< 0.01
Gamma-glutamyl transferase	11.17a	14.67a	15.60a	12.50a	23.80b	21.67b	22.50b	0.86	< 0.01
Aspartate amino transferase	297.18a	311.25ab	324.38ab	285.65a	277.77a	326.08ab	379.26b	7.85	< 0.01
Alkaline phosphatase	1389.33	1308.50	1312.67	1308.67	1374.67	1323.50	1305.00	11.46	0.24

*SEM* Standard error of the mean, *T1* Corn-soybean-based basal ration, *T2* Ration containing 15% unheated HSC, *T3* Ration containing 15% unheated HSC and 1000 FTU of phytase, *T4* Ration containing 15% autoclaved HSC (120 °C, 10 min), *T5* Ration containing 15% autoclaved HSC (120 °C, 10 min) and 1000 FTU of phytase, *T6* Ration containing 15% oven-heated HSC (120 °C, 20 min), *T7* Ration containing 15% oven-heated HSC (120 °C, 20 min) and 1000 FTU of phytase. Different superscripts in the same row are significantly different at *P* < 0.05 and very significantly different at *P* < 0.01. Each value is the mean of 6 replicates of each treatment (one bird per replicate)

The increase in serum gamma-glutamyl transferase and aspartate aminotransferase fed oven-heated HSC diets may be due to liver damage caused by acrylamide. High gamma-glutamyl transferase and aspartate aminotransferase are indicators of liver damage (Lai et al. 2024), and acrylamide is a harmful by-product formed during the Maillard reaction through the degradation of amino acids, especially asparagine, in the presence of sugars (Augustine and Bent 2022). Acrylamide also increased the hepatic oxidative stress markers level, including lipid peroxides and protein carbonyl content (Zhang et al. 2023a, b). These conditions may have contributed to the potential increase in gamma-glutamyl transferase levels in the blood of broilers fed oven-heated HSC. Gamma-glutamyl transferase is an enzyme mainly found in the liver and is involved in glutathione metabolism, which is very important as an antioxidant (Xing et al. 2022).

The heating process decreased serum total protein and serum albumin of broiler chickens. The decrease in total serum protein may be due to proteins and amino acids that are sensitive to the heating process. The structural integrity of proteins may be compromised by exposure to heat during processing, which may lead to denaturation (Nahavandinejad et al. 2014), which in turn may result in reduced serum albumin levels. Heating feed ingredients at the right temperature and time can improve nutrient digestibility and reduce anti-nutrients. On the other hand, increasing temperature and duration negatively affect nutrient content and digestibility. For instance, the energy and amino acid digestibility of soybean and canola meal decreased when autoclaved at 125–150 °C for 30 min, where the heat-induced decrease in amino acid digestibility was mainly due to the Maillard reaction (Oliveira et al. 2019) between the carbonyl group of the reducing sugar and free amino group (Haryanti et al. 2020). Furthermore, lysine is the amino acid that is most susceptible to Maillard reactions due to the presence of ε-amino groups (Eklund et al. 2015).

Autoclaved HSC (120 °C, 10 min) increased serum glucose levels compared to the unheated HSC and control group. Autoclaving with steam and pressure is presumably more effective than oven heating in gelatinizing starch, making it easier to be digested and absorbed by the intestines of broilers (Wang et al. 2023). Moreover, starch gelatinization has a direct effect on starch digestibility due to the destruction of glycosidic bonds, rendering the molecules susceptible to digestive enzymes. Additionally, gelatinization increases the amylopectin content and reduces the amylose content, thereby enhancing digestibility (Ali et al. 2020). This increased starch digestibility contributes to increased blood glucose levels that can be used for energy and growth of broilers, as evidenced by higher body weight production in the autoclaved HSC group compared to the unheated and oven-heated groups.

In our study, the addition of phytase to autoclaved HSC appeared to be more effective in increasing serum calcium levels compared to unheated HSC. This is in line with previous studies that increased Ca digestibility in broiler chickens fed autoclaved meat and bone meal at 128 °C for 90 min (Zanu et al. 2021). Meanwhile, the phytase enzyme helps to unbind phytates of minerals, including calcium. As a result, heat-processed HSCs with phytase enzymes can release calcium more efficiently during digestion, which in turn improves absorption and subsequently increases blood calcium levels. This further emphasizes that autoclave heating and phytase enzyme addition can reduce the formation of calcium-phytate complexes and increase calcium absorption.

Our findings suggest that heating treatment can reduce the anti-nutrient content of HSC. Dietary autoclaved HSC decreases *E. coli* and increases *L. acidophilus* in the cecum, intestinal villi height, calcium, and glucose in serum, and yields better body weight and weight gain of broiler chicken than oven-heated HSC and unheated HSC. However, the positive effect of dietary autoclaved HSC on broiler performance cannot match that of soybean meal and corn-based diets. Dietary 15% HSC decreases feed intake and enlarges the ileum, jejunum, and cecum weight. Meanwhile, the beneficial effect of phytase is more prominent when added to unheated HSC. Future research should focus on optimizing the autoclaved HSC and developing strategies to reduce its crude fiber content to improve digestibility and broiler performance.

## Data Availability

The datasets created or analyzed in our study are available from the corresponding author upon reasonable request.

## References

[CR1] Abun A, Prasetya AH, Widjastuti T (2024) The effect of Lacto-B probiotics on broiler chicken performance. World J Adv Res Rev 21:1670–1677

[CR2] Adekanmi OK, Oluwatooyin OF, Yemisi AA (2009) Influence of processing techniques on the nutrients and antinutrients of Tigernut (Cyperus esculentus L.). World J Dairy Food Sci 4:88–93

[CR3] Al-Harthi MA, Attia YA, Elgandy MF, Bovera F (2023) The effects of *Moringa peregrina* seed meal, autoclaving, and/or exogenous enzyme cocktail on performance, carcass traits, meat quality, and blood lipids of broilers. Front Vet Sci 10:1–18 10.3389/fvets.2023.1158468 PMC10354260 37476825

[CR4] Ali S, Singh B, Sharma S (2020) Effect of processing temperature on morphology, crystallinity, functional properties, and in vitro digestibility of extruded corn and potato starches. J Food Process Preserv 44:1–8

[CR5] Association of Official Analytical Chemists (AOAC) (1990) Methods of analysis of the association of official analytical chemists, 18th edn. Association of Official Analytical Chemists, Washington

[CR6] Augustine DA, Bent G (2022) Acrylamide, a toxic maillard by-product and its inhibition by sulfur-containing compounds: a mini-review. Front Food Sci Technol 2:1–7

[CR7] Badaruddin R, Auza FA, Syamsuddin Nafiu LO, Saili T, Pagala MA, Munadi LO (2022) Percentage of internal organs of broiler chickens given Vernonia amygdalina flour feed additives. IOP Conf Ser Earth Environ Sci 1107:012069

[CR8] Boroojeni FG, Vahjen W, Mader A, Knorr F, Ruhnke I, Röhe I, Hafeez A, Villodre C, Männer K, Zentek J (2014) The effects of different thermal treatments and organic acid levels in feed on microbial composition and activity in gastrointestinal tract of broilers. Poult Sci 93:1440–1452 24879694 10.3382/ps.2013-03763

[CR9] Dundar AN, Gocmen D (2013) Effects of autoclaving temperature and storing time on resistant starch formation and its functional and physicochemical properties. Carbohydr Polym 97:764–771 23911513 10.1016/j.carbpol.2013.04.083

[CR10] Eizadi E, Shariatmadari F, Karimi Torshizi MA, Matin HR (2015) Broiler chicken performance in response to various levels of raw and autoclaved rice bran. Bulg J Vet Med 18:348–360

[CR11] Eklund M, Sauer N, Schöne F, Messerschmidt U, Rosenfelder P, Htoo JK, Mosenthin R (2015) Effect of processing of rapeseed under defined conditions in a pilot plant on chemical composition and standardized ileal amino acid digestibility in rapeseed meal for pigs. J Anim Sci 93:2813–2825 26115269 10.2527/jas.2014-8210

[CR12] Erener G, Ocak N, Ozturk E, Cankaya S, Ozkanca R, Altop A (2020) Evaluation of olive leaf extract as a growth promoter on the performance, blood biochemical parameters, and caecal microflora of broiler chickens. Rev Bras Zootec 49:e20180300

[CR13] Feng X, Sun G, Fang Z (2022) Effect of hempseed cake (Cannabis sativa L.) incorporation on the physicochemical and antioxidant properties of reconstructed potato chips. Foods 11:211 35053943 10.3390/foods11020211 PMC8775051

[CR14] Geng L, Liu K, Zhang H (2023) Lipid oxidation in foods and its implications on proteins. Front Nutr 10:1–12 10.3389/fnut.2023.1192199 PMC10307983 37396138

[CR15] Haetinger VS, Adeola O (2023) Regression-derived phosphorus digestibility responses of broiler chickens to heat treatment of soybean meal and poultry meal. Poult Sci 102:102299 36436370 10.1016/j.psj.2022.102299 PMC9706607

[CR16] Haryanti P, Supriyadi MDW, Santoso U (2020) The changes of chemical composition and antioxidant activity of coconut sap during heating process. Rasayan J Chem 13:2010–2019

[CR17] Iji PA, Hughes RJ, Choet M, Tivey RR (2001) Intestinal structure and function of broiler chickens on wheat-based diets supplemented with a microbial enzyme. Asian-Aust J Anim Sci 14:54–60

[CR18] Jha R, Mishra P (2021) Dietary fiber in poultry nutrition and their effects on nutrient utilization, performance, gut health, and on the environment: a review. J Anim Sci Biotechnol 12:1–16 33866972 10.1186/s40104-021-00576-0 PMC8054369

[CR19] Kasula R, Solis F, Shaffer B, Connett F, Barrett C, Cocker R, Wellinghan E (2021) Effect of dietary hemp seed cake on the performance of commercial laying hens. Int J Livest Prod 12:17–27

[CR20] Khan RU, Durrani FR, Chand N, Anwar H (2010) Influence of feed supplementation with *cannabis sativa* on quality of broilers carcass. Pak Vet J 30:34–38

[CR21] Konca Y, Yuksel T, Yalcin H, Beyzi SB, Kaliber M (2019) Effects of heat-treated hempseed supplementation on performance, egg quality, sensory evaluation and antioxidant activity of laying hens. Br Poult Sci 60:39–46 30421987 10.1080/00071668.2018.1547360

[CR22] Krieg J, Borda-Molina D, Siegert W, Sommerfeld V, Chi YP, Taheri HR, Feuerstein D, Camarinha-Silva A, Rodehutscord M (2021) Effects of calcium level and source, formic acid, and phytase on phytate degradation and the microbiota in the digestive tract of broiler chickens. Anim Microbiome 3:23 33722307 10.1186/s42523-021-00083-7 PMC7962351

[CR23] Kriseldi R, Johnson JA, Walk CL, Bedford MR, Dozier WA (2021) Influence of exogenous phytase supplementation on phytate degradation, plasma inositol, alkaline phosphatase, and glucose concentrations of broilers at 28 days of age. Poult Sci 100:224–234 33357685 10.1016/j.psj.2020.10.004 PMC7772694

[CR24] Kuku A, Etti U, Ibironke I (2014) Processing of fluted pumpkin seeds, *Telfairia occidentalis* (hook f) as it affects growth performance and nutrient metabolism in rats. African J Food Agric Nutr Dev 14:192–214

[CR25] Kumar I, Bhattacharya J, Das BK, Lahiri P (2020) Growth, serum biochemical, and histopathological responses of broilers administered with silver nanoparticles as a drinking water disinfectant. Biotech 10:1–12 32099735 10.1007/s13205-020-2101-1 PMC7002811

[CR26] Lai X, Chen H, Dong X, Zhou G, Liang D, Xu F, Liu H, Luo Y, Liu H, Wan S (2024) AST to ALT ratio as a prospective risk predictor for liver cirrhosis in patients with chronic HBV infection. Eur J Gastroenterol Hepatol 36:338–344 38251454 10.1097/MEG.0000000000002708 PMC10833202

[CR27] Nahavandinejad M, Seidavi A, Asadpour L, Payan-Carreira R (2014) Blood biochemical parameters of broilers fed differently thermal processed soybean meal. Rev MVZ Cordoba 19:4301–4315

[CR28] Nari N, Ghasemi HA, Hajkhodadadi I, Farahani AHK (2019) Intestinal microbial ecology, immune response, stress indicators, and gut morphology of male broiler chickens fed low-phosphorus diets supplemented with phytase, butyric acid, or *Saccharomyces boulardii*. Livest Sci 234:103975

[CR29] National Research Council (1994) Nutrient requirements of poultry, 9th Edn. National Academy Press. 157

[CR30] Oliveira MSF, Wiltafsky MK, Lee SA, Kwon WB, Stein HH (2019) Concentrations of digestible and metabolizable energy and amino acid digestibility by growing pigs may be reduced by autoclaving soybean meal. Anim Feed Sci Technol 269:114621

[CR31] Omotoso AO, Reyer H, Oster M, Ponsuksili S, Wimmers K (2023) Jejunal microbiota of broilers fed varying levels of mineral phosphorus. Poult Sci 102:103096 37797492 10.1016/j.psj.2023.103096 PMC10562922

[CR32] Ondrej S, Fılıp K, Hana S, Vaclav, T, Tomas V, Leos P, Eva M (2015) The effect of hempseed cakes on broiler chıckens performance parameters. Mandelnet. 157–160

[CR33] Öztürk E, Darmawan A, Özlü Ş, Abacı SH (2024) Effects of dietary local hemp seed meal as soybean meal alternative on productive performance egg quality and yolk fatty acid composition of laying hens. Arch Anim Nutr 78(2):177–190 10.1080/1745039X.2024.2373485 39047154

[CR34] Ptak A, Bedford MR, wia¸tkiewicz S, Zyła K, Józefiak D (2015) Phytase modulates ileal microbiota and enhances growth performance of the broiler chickens. PLoS One 10:1–15 10.1371/journal.pone.0119770 PMC4363628 25781608

[CR35] Rehman ZU, Shah WH (2005) Thermal heat processing effects on antinutrients, protein and starch digestibility of food legumes. Food Chem 91(2):327–331

[CR36] Russo R, Reggiani R (2015) Evaluation of protein concentration, amino acid profile and anti-nutritional compounds in hempseed meal from dioecious and monoecious varieties. Am J Plant Sci 6:14–22

[CR37] Semwogerere F, Katiyatiya CLF, Chikwanha OC, Marufu MC, Mapiye C (2020) Bioavailability and bioefficacy of hemp by-products in ruminant meat production and preservation: a review. Front Vet Sci 7:572906 33102571 10.3389/fvets.2020.572906 PMC7545362

[CR38] Sittiya J, Yamauchi K, Nimanong W, Thongwittaya N (2020) Influence of levels of dietary fiber sources on the performance, carcass traits, gastrointestinal tract development, fecal ammonia nitrogen, and intestinal morphology of broilers. Brazilian J Poult Sci 22:001–008

[CR39] Šťastník O, Jůzl M, Karásek F, Fernandová D, Mrkvicová E, Pavlata L, Nedomová S, Vyhnánek T, Trojan V, Doležal P (2019) The effect of hempseed expellers on selected quality indicators of broiler chicken’s meat. Acta Vet Brno 88:121–128

[CR40] Tejeda JO, Kim W (2021) Role of dietary fiber in poultry nutrition. Animals 11:461 33572459 10.3390/ani11020461 PMC7916228

[CR41] Tufarelli V, Losacco C, Tedone L, Passantino L, Tarricone S, Laudadio V, Colonna MA (2023) Hemp seed (*Cannabis sativa* L.) cake as a sustainable dietary additive in slow-growing broilers: effects on performance, meat quality, oxidative stability and gut health. Vet Quart 43:1–12 10.1080/01652176.2023.2260448 PMC10524784 37715944

[CR42] Wang Q, Xiong YL (2019) Processing, nutrition, and functionality of hempseed protein: a review. Compr Rev Food Sci Food Saf 18:936 33336999 10.1111/1541-4337.12450

[CR43] Wang X, Du B, Nian F, Ru Y, Sun L, Qin S, Qin S, Tang D (2023) Effects of processing methods and conditioning temperatures on the cassava starch digestibility and growth performance of broilers. Animals 13:1373 37106936 10.3390/ani13081373 PMC10134972

[CR44] Xing M, Gao M, Li J, Han P, Mei L, Zhao L (2022) Characteristics of peripheral blood gamma-glutamyl transferase in different liver diseases. Medicine 101:E28443 35029891 10.1097/MD.0000000000028443 PMC8735790

[CR45] Zanu HK, Kheravii SK, Morgan NK, Bedford MR, Swick RA (2021) Over-processed meat and bone meal and phytase effects on broilers challenged with subclinical necrotic enteritis: Part 3. Bone mineralization and litter quality. Anim Nutr 7:142–151 33997342 10.1016/j.aninu.2020.06.007 PMC8110868

[CR46] Zhang L, Yang L, Luo Y, Dong L, Chen F (2023a) Acrylamide-induced hepatotoxicity through oxidative stress: mechanisms and interventions. Antioxid Redox Signal 38:1122–1137 36322716 10.1089/ars.2022.0055

[CR47] Zhang C, Hao E, Chen X, Huang C, Liu G, Chen H, Wang D, Shi L, Xuan F, Chang D, Chen Y (2023b) Dietary fiber level improves growth performance, nutrient digestibility, immune and intestinal morphology of broilers from day 22 to 42. Animals 13:1227 37048483 10.3390/ani13071227 PMC10093110

